# Sex-Related Differences in Oxidative, Platelet, and Vascular Function in Chronic Users of Heat-not-Burn vs. Traditional Combustion Cigarettes

**DOI:** 10.3390/antiox11071237

**Published:** 2022-06-24

**Authors:** Leonardo Schirone, Lorenzo Loffredo, Roberto Carnevale, Simona Battaglia, Roberta Marti, Stefano Pizzolo, Simona Bartimoccia, Cristina Nocella, Vittoria Cammisotto, Wael Saade, Alessandra Tanzilli, Sebastiano Sciarretta, Isotta Chimenti, Elena De Falco, Elena Cavarretta, Vittorio Picchio, Mariangela Peruzzi, Antonino Marullo, Fabio Miraldi, Francesco Violi, Andrea Morelli, Giuseppe Biondi-Zoccai, Giacomo Frati

**Affiliations:** 1 Department of Medical-Surgical Sciences and Biotechnologies, Sapienza University of Rome, Corso della Repubblica 79, 04100 Latina, Italy; roberto.carnevale@uniroma1.it (R.C.); sebastiano.sciarretta@uniroma1.it (S.S.); isotta.chimenti@uniroma1.it (I.C.); elena.defalco@uniroma1.it (E.D.F.); elena.cavarretta@uniroma1.it (E.C.); vittorio.picchio@uniroma1.it (V.P.); antonino.marullo@uniroma1.it (A.M.); giuseppe.biondizoccai@uniroma1.it (G.B.-Z.); giacomo.frati@uniroma1.it (G.F.); 2 Department of Clinical Internal, Anesthesiologic and Cardiovascular Sciences, Sapienza University of Rome, Viale del Policlinico 155, 00161 Rome, Italy; lorenzo.loffredo@uniroma1.it (L.L.); simona.bartimoccia@uniroma1.it (S.B.); cristina.nocella@uniroma1.it (C.N.); vittoria.cammisotto@uniroma1.it (V.C.); wael.saade@uniroma1.it (W.S.); alessandra.tanzilli@uniroma1.it (A.T.); mariangela.peruzzi@uniroma1.it (M.P.); fabio.miraldi@uniroma1.it (F.M.); francesco.violi@uniroma1.it (F.V.); andrea.morelli@uniroma1.it (A.M.); 3 Mediterranea Cardiocentro, Via Orazio 2, 80122 Napoli, Italy; 4 Department of Molecular Medicine, Sapienza University of Rome, Viale del Policlinico 155, 00161 Rome, Italy; simona.battaglia@uniroma1.it (S.B.); roberta.marti@uniroma1.it (R.M.); stefano.pizzolo@uniroma1.it (S.P.); 5 IRCCS NeuroMed, Via Atinense 18, 86077 Pozzilli, Italy

**Keywords:** cardiovascular disease, heat-not-burn cigarette, modified risk product, sex, smoking

## Abstract

Smoking is still a major cardiovascular risk factor, despite many public awareness campaigns and dedicated interventions. Recently, modified risk products (MRP), e.g., heat-not-burn cigarettes (HNBCs), have been introduced as surrogates of traditional combustion cigarettes (TCCs). Although these products are promoted as healthier than TCCs, few studies have been conducted to assess it. This work is a sex-focused sub-study of a prospective observational study in which apparently healthy chronic TCC smokers were age-matched with regular HNBC users. Blood samples were collected for biochemical assays and blood pressure and flow-mediated dilation (FMD) were measured. Out of 60 subjects, 33 (55%) were women, and 27 (45%) men, with 11 (33%) vs. 9 (33%) non-smokers, respectively, 10 (30%) vs. 10 (37%) TCC smokers, and 12 (36%) vs. 8 (30%) HNBC smokers (*p* = 0.946). Bivariate and multivariable analyses showed no statistically significant between-sex differences in NO, H_2_O_2_, sCD40L, sNox2-dp, sP-selectin, platelet aggregation, cotinine or FMD, overall, in non-smokers, in TCC smokers, or in HNBC smokers (all *p* > 0.05). HNBCs appeared safer than TCCs when focusing on Nox2-dp (*p* = 0.026) and sP-selectin (*p* = 0.050) but had similar levels of the other measured markers. In conclusion, HNBCs have similar detrimental effects on women and men’s oxidative stress (H_2_O_2_: *p* = 0.49; sNox2-dp: *p* = 0.31) and platelet activation (sP-selectin: *p* = 0.33; platelet aggregation *p* = 0.87).

## 1. Introduction

Smoking is a highly addictive habit that can lead to increased morbidity and reduced life expectancy due to atherothrombotic cardiovascular diseases, obstructive pulmonary disease and cancer, as well as many other conditions [1,2]. In fact, a high-temperature aerosol is produced by the combustion of tobacco, releasing thousands of toxicologically significant chemicals, including heavy metals (e.g., lead, cadmium, arsenic), aromatic hydrocarbons (e.g., toluene, phenols, benzopyrene), and many other organic toxins (e.g., carbon monoxide, hydrogen cyanide, and formaldehyde) [3].

In the past, much effort was put into identifying effective strategies to enable cessation and effective abstinence from traditional combustion cigarettes (TCCs), ranging from nicotine replacement therapies to modified risk products (MRPs), e.g., electronic vaping cigarettes (EVCs) and heat-not-burn cigarettes (HNBCs) [4,5,6,7,8,9]. Indeed, MRPs have substantial potential in supporting smokers to discontinue TCCs, with the hopeful eventual abstinence from smoking any tobacco-related product by scalar reduction of nicotine dosage while maintaining the behavioral aspects of the habit [10,11,12]. However, MRPs may often chronically replace TCCs, thus still exposing the patients to a smoldering but clinically relevant increased cardiovascular risk compared to non-smokers [13]. Last, MRPs could be very attractive for adolescents and young adults, representing a bad habit that increases the risk of transition to start smoking TCCs.

While MRPs have been approved and commercialized for human use, and some advocate their reimbursement as a cigarette-discontinuation strategy, the actual safety of these products is still a matter of debate [14,15], and their sex-specific effects remain to be appraised in detail. Indeed, sex is a significant contributor or modulator of cardiovascular, pulmonary, and neoplastic risk. Moreover, nicotine pharmacokinetics, pharmacodynamics, inflammatory response, pulmonary toxicity and neurotoxicity are different in males and females [16,17]. Thus, it is essential to attentively gauge the potential interacting role of sex with the toxicity of chronic HNBC use [18]. 

We aimed to appraise the potential modulating role of sex in a recently completed case-control study on the impact of chronic TCC vs. HNBC use, focusing on oxidative, platelet aggregation, and vascular parameters.

## 2. Materials and Methods

### 2.1. Design

The present work is a sub-study focusing on sex of the Sapienza University of Rome-Vascular Assessment of Proatherosclerotic Effects of Smoking (SUR-VAPES) Chronic study, which has been reported in detail elsewhere [19]. Briefly, the SUR-VAPES Chronic study was an observational study conducted between September 2019 and January 2020, including 20 chronic (>1 month) users of heat-not-burn cigarettes (HNBCs), 20 chronic (>1 month) smokers of traditional combustion cigarettes (TCCs), and 20 non-smokers. The subjects were volunteer nurses, trainees, and blood donors, and all provided written informed consent. All HNBCs smokers were previously TCCs users and had no longer smoked these for a mean of 1.5 ± 0.5 years. Participants took neither vitamin E (or other antioxidants) nor anti-platelet drugs in the month preceding and during the study. Female patients included in the study were not menstruating during the study procedures. The subjects were included if considered healthy (i.e., no acute or chronic metabolic, inflammatory, or organ disease; no fever or infection in the past 3 months; no allergies; no cardiovascular symptoms; normal cardiovascular pression and heart rhythm at screening). Blood samples collection and FMD were performed at 8 a.m. after 8 h of abstinence from smoking and eating. The study was conducted following the principles of the Declaration of Helsinki and approved by the local ethical committee (Protocol Number 813/14).

### 2.2. Flow-Mediated Dilation

Endothelial-dependent flow-mediated dilation (FMD) and basal brachial artery diameter were assessed by ultrasonography according to established means [19]. FMD was measured between 8 a.m. and 10 a.m. on days 1–7 in subjects resting supine in a temperature-controlled room (22.8 °C). A 7.5-MHz linear array transducer ultrasound system (Samsung HS30, Samsung, Seoul, Korea) equipped with electronic calipers was used to measure the brachial artery 3–7 cm above the antecubital crease. A vascular software was used to display two-dimensional imaging, color/spectral Doppler and internal electrocardiogram. A sphygmomanometric cuff was placed on the forearm and inflated 50 mm Hg above systolic pressure for 5 min to occlude brachial artery inflow and create a flow stimulus in the vessel. Vasodilation was measured at the end of diastole and FMD was expressed as a percentage of the baseline diameter observed after artery occlusion. The operator was blinded to the patients’ study assignment.

### 2.3. Laboratory Analyses

Blood draws were performed using collection tubes with or without 3.8% sodium citrate, based on centrifugation for 10 min at 300× *g*. As specified below, the obtained supernatants were immediately frozen and kept at −80 °C until use. Unless otherwise detailed, all materials were purchased from Sigma-Aldrich/Merck (St. Louis, MO, USA).

#### 2.3.1. Plasma Soluble CD40 Ligand Assay

The plasmatic levels of soluble CD40 ligand (sCD40L) were measured by a commercial enzymatic kit (Cusabio, Houston, TX, USA). Values were expressed as ng/mL. Intra-assay coefficient of variability (CV) was <8%, inter-assay CV was <10%.

#### 2.3.2. Plasma Soluble P-Selectin

The plasmatic concentration of soluble P-selectin (sP-selectin) was assessed by a commercial ELISA kit (Cusabio, Houston, TX, USA). Values were expressed as ng/mL. Intra-assay CV was <8%, inter-assay CV was <10%.

#### 2.3.3. Platelet Preparation and Aggregation

Platelet-rich plasma (PRP) samples were obtained from blood collected in 3.8% sodium citrate after 15 min of centrifugation at 180 g and only the upper 75% supernatant was collected to avoid leukocyte contamination. Then, 2 µg/mL collagen (Mascia Brunelli, Milan, Italy) were added, and the samples were incubated for 10 min at 37 °C. Platelet aggregation was assessed in siliconized glass cuvettes under stirring conditions at the rate of 1200 rpm, and data were acquired in 2 dual-channel modules (Chrono-Log Model 700, Chrono-Log, Havertown, PA, USA) light transmission aggregometer.

#### 2.3.4. Serum Cotinine

The serum concentration of cotinine, a primary biomarker for the assessment of tobacco exposure, was measured by a commercial ELISA kit (Origene, Rockville, MD, USA). Values were expressed as ng/mL. The intra-assay and inter-assay CV were <10%.

#### 2.3.5. Serum Nitric Oxide Bioavailability

The serum bioavailability of nitric oxide (NO) was assessed by a colorimetric kit (Arbor Assays, Ann Arbor, MI, USA). Values were expressed as µM. The intra-assay CV was 6.8%, and the inter-assay CV was 7.4%.

#### 2.3.6. Serum Hydrogen Peroxide Production

Serum levels of hydrogen peroxide (H_2_O_2_) were measured through a commercial colorimetric kit (Arbor Assays), and values were expressed as µM. The intra-assay CV was 2.1%, and the inter-assay CV was 3.7%.

#### 2.3.7. Serum Soluble Nox2-Derived Peptide Assay

Serum levels of Nox2-derived peptide (sNox2-dp) were detected by a custom ELISA method as previously described [20]. Values were expressed as pg/mL. The intra-assay CV was 5.2%, and the inter-assay CV was 6%.

### 2.4. Statistical Analysis

Continuous variables are reported as mean ± standard deviation and categorical variables as count (%). Baseline comparisons were based on the unpaired Student *t*-test for continuous variables and Fisher exact test for categorical variables. The impact of sex in the overall study sample and its interaction with smoking status (TCCs, HNBCs, none) was tested with a multivariable linear regression model. Data were assessed for normal distribution by visual inspection of the box plots. Statistical significance was set at a 2-tailed 0.05, without multiplicity adjustment. Computations were performed with Stata 13 (StataCorp, College Station, TX, USA). 

## 3. Results

The study was conducted on 20 chronic users of HNBCs, 20 regular smokers of TCCs, and 20 non-smokers. 

Out of 60 subjects, 33 (55%) were women and 27 (45%) men. Out of these fractions, 11 (33%) vs. 9 (33%) were non-smokers, 10 (30%) vs. 10 (37%) TCCs smokers, and 12 (36%) vs. 8 (30%) HNBCs smokers (*p* = 0.946) (Table 1).

Since this study was intended to unveil sex-related differences in terms of oxidative stress, platelet and vascular function in chronic users of heat-not-burn or combustion cigarettes, we first performed a bivariate analysis followed by a multivariable analysis.

**Figure 1:**
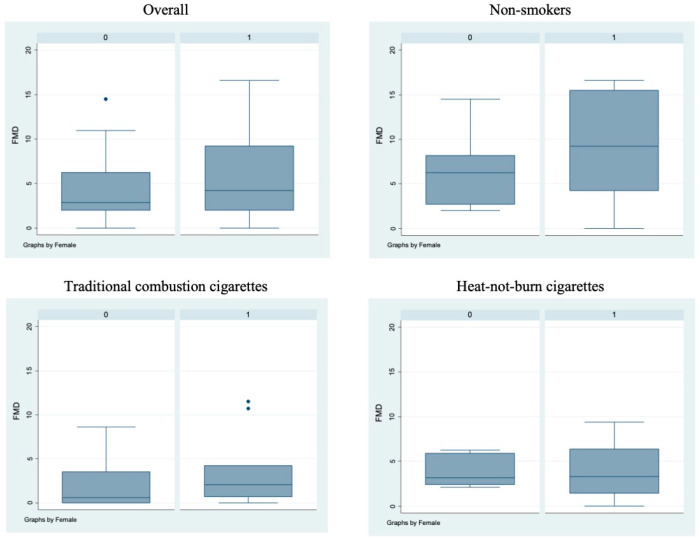
Flow-mediated dilation values in the different sub-populations. Box-and-whisker plots representing flow-mediated dilation values (%) in the overall cohort and in non-smokers, TCCs-smokers and HNBCs-smokers subgroups. 0 = males; 1 = females; • = outlier sample.

**Figure 2:**
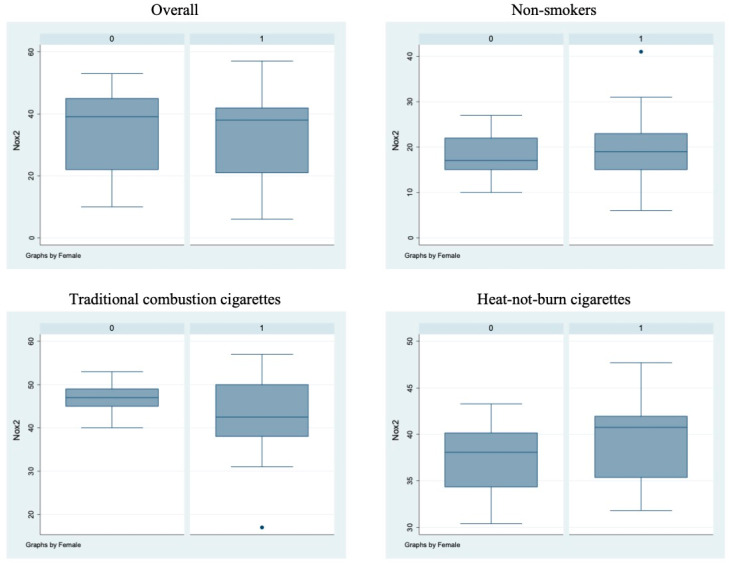
sNox2-dp circulating levels in the different sub-populations. Box-and-whisker plots representing sNox2-dp circulating levels (ng/mL) in the overall cohort and in non-smokers, TCCs-smokers and HNBCs-smokers subgroups. 0 = males; 1 = females; • = outlier sample.

**Figure 3:**
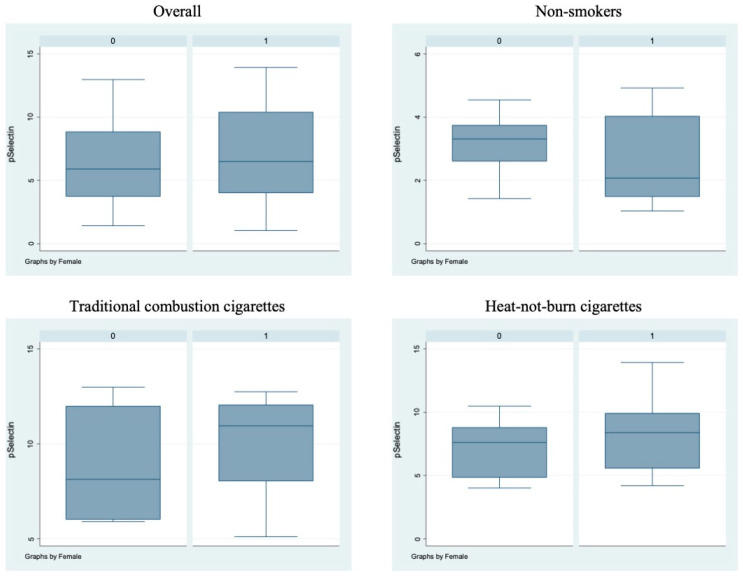
sP-selectin circulating levels in the different sub-populations. Box-and-whisker plots representing sP-selectin circulating levels (ng/mL) in the overall cohort and in non-smokers, TCCs-smokers and HNBCs-smokers subgroups. 0 = males; 1 = females.

Bivariate analysis showed no statistically significant differences (*p* > 0.05) between male and female subjects in FMD (Figure 1), NO, H_2_O_2_, sCD40L, Nox2-dp (Figure 2), P-selectin (Figure 3), platelet aggregation, or cotinine parameters among the three examined groups, i.e., non-smokers, TCCs smokers, and HNBCs smokers (Table 2, Table 3 and Table 4). These results suggest that sex does not impact the detrimental effects of smoking TCCs or HNBCs.

Similarly, we performed a multivariable analysis that did not show any significant sex-related difference, either overall or at interaction testing. Notably, TCCs and HNBCs appeared similarly detrimental on NO, H_2_O_2_, sCD40L, platelet aggregation, cotinine, and FMD, whereas HNBCs appeared less harmful than TCCs when focusing on Nox2-dp (*p* = 0.026) and P-selectin (*p* = 0.050) (Table 5).

We concluded that smoking HNBCs or TCCs has a similar negative impact on reactive oxygen species (ROS) generation and vascular reactivity, although HNBC smokers display lower levels of markers related to platelet activation.

## 4. Discussion

The past decade has marked a significant evolution of the tobacco market in the US and Europe, with an overall decrease in the use of cigarettes coupled with increased use of MRPs. 

However, in this regard, research fails to provide differences in the use and, more importantly, in the spectrum of detrimental effects of TCCs and MRPs among specific groups, particularly women vs. men. The current study fills the gap in the current literature by examining for the first time the potential interacting role of sex with the detrimental effects of chronic HNBC use. 

**Figure 4:**
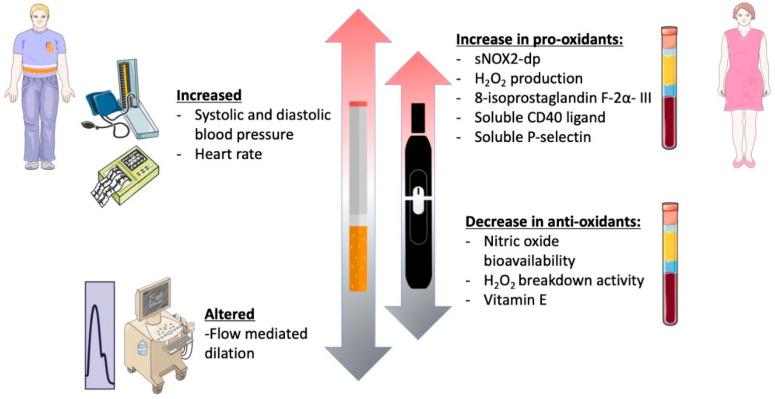
Graphical abstract of the study.

In this prospective observational cross-sectional study, chronic smoking of both TCCs and HNBCs was associated with reduced endothelial function, increased oxidative stress, and platelet activation, in accordance with previous studies (Figure 4) [19,21,22,23]. However, since this was a sex-focused sub-study intended to unveil sex-related differences concerning different smoking habits, we deeply analyzed gender-related differences with respect to redox state, platelet aggregation, and vascular function. In this setting, smoking HNBCs or TCCs showed similar detrimental effects in women and men, in contrast with the current knowledge on the impact of traditional smoking on both cardiovascular and pulmonary health, which seems to be more severe in females [24,25,26]. However, the discrepancy found in our results may be partially explained by the lower mean age of the patients enrolled for the study, which is below 40 years, and, consequently, by the relatively few years of smoking habit. In fact, within the TCCs group, male patients had been smoking for 4.5 years on average, while females for 8.2 years. Moreover, within the HNBCs group, both males and females had been smoking for less than 2 years on average. This is not surprising and most likely because HNBCs have been commercially available in Italy quite recently; indeed, it is conceivable that some previous chronic TCC smokers may have recently switched to HNBC use for health reasons.

## 5. Conclusions

In our study, HNBCs and TCCs have similar detrimental effects on women and men’s oxidative stress and platelet activation. Nevertheless, we cannot exclude that smoking HNBCs for more extended periods may unveil sex-related differences in the development of cardiovascular pathologies. In fact, increasing evidence highlights that sex highly impacts the body’s response to exogenous bioactive agents and chemicals [27]. Integrating sex-based omics studies might help develop more effective and accurate diagnostic tools and personalized therapies to improve the spectrum of parameters taken into consideration by the so-called “precision medicine”. Thus, in the field examined in this study, prospective future randomized controlled trials are needed to analyze the long-term relationship between endothelial function, oxidative stress, and cardiovascular events in male and female HNBC users. Accordingly, since this was a small pilot study based on health professionals, we aim for the future to consider larger cohorts representative of the general population and enroll patients with a longer history of smoking habits of HNBCs.

## Figures and Tables

**Table 1:** Overall cohort.

Characteristic	Male	Female	*p*
Subjects	27	33	-
Age (years)	30.2 ± 8.5	30.8 ± 7.7	0.763
Height (cm)	178.3 ± 6.4	165.9 ± 7.5	<0.001
Weight (kg)	77.4 ± 8.9	58.4 ± 7.1	<0.001
Body mass index (kg/m^2^)	24.4 ± 2.7	21.3 ± 2.5	<0.001
Systolic blood pressure (mmHg)	123.2 ± 14.3	112.4 ± 8.9	<0.001
Diastolic blood pressure (mmHg)	77.5 ± 7.2	71.4 ± 9.4	0.007
Mean blood pressure (mmHg)	92.7 ± 6.6	85.1 ± 8.4	<0.001
Total cholesterol (mg/dL)	180.0 ± 14.2	174.7 ± 14.5	0.163
Smoking status			0.946
Non-smoker	9 (33.3%)	11 (33.3%)	
Traditional combustion cigarette smoker	10 (37.0%)	10 (30.3%)	
Heat-not-burn cigarette smoker	8 (29.6%)	12 (36.4%)	
Years of smoking	2.2 ± 2.2	3.0 ± 4.0	0.349
Cigarettes per day	9.8 ± 9.3	8.0 ± 6.6	0.390
Flow-mediated dilation (%)	4.0 ± 3.6	5.5 ± 5.0	0.198
Nitric oxide (µmol/L)	20.8 ± 15.8	22.1 ± 18.6	0.767
sNox2-dp (ng/mL)	34.3 ± 13.0	33.9 ± 12.9	0.887
H_2_O_2_ (µmol/L)	25.6 ± 15.7	22.8 ± 16.7	0.519
sCD40L (ng/mL)	2.5 ± 1.1	2.7 ± 1.2	0.322
sP-selectin (ng/mL)	6.4 ± 3.2	7.0 ± 3.9	0.584
Platelet aggregation (%)	71.3 ± 12.4	73.4 ± 10.4	0.465
Cotinine (ng/mL)	95.5 ± 78.5	94.2 ± 72.0	0.949

H_2_O_2_ = serum hydrogen peroxide production; NO = serum nitric oxide bioavailability; sCD40L = plasmatic levels of soluble CD40 ligand; sNox2-dp = serum soluble Nox2-derived peptide; sP-selectin = plasmatic concentration of soluble P-selectin.

**Table 2:** Non-smokers.

Characteristic	Male	Female	*p*
Subjects	9	11	-
Age (years)	30.0 ± 6.5	27.6 ± 5.2	0.380
Height (cm)	175 ± 6.6	161 ± 6.8	<0.001
Weight (kg)	76.3 ± 5.9	57.6 ± 8.1	<0.001
Body mass index (kg/m^2^)	25.0 ± 2.4	22.1 ± 2.3	<0.05
Systolic blood pressure (mmHg)	130 ± 20.9	111.2 ± 7.2	<0.05
Diastolic blood pressure (mmHg)	78.9 ± 8.2	68.2 ± 10.6	<0.05
Mean blood pressure (mmHg)	95.9 ± 8.2	82.5 ± 9.0	<0.01
Total cholesterol (mg/dL)	181.1 ± 9.4	176.4 ± 16.7	<0.459
Flow-mediated dilation (%)	6.3 ± 4.4	9.1 ± 5.7	0.244
Nitric oxide (µmol/L)	41.4 ± 8.3	46.1 ± 11.8	0.333
sNox2-dp (ng/mL)	18.2 ± 5.3	20.4 ± 9.4	0.550
H_2_O_2_ (µmol/L)	9.7 ± 3.8	8.4 ± 3.6	0.434
sCD40L (ng/mL)	1.4 ± 0.5	1.6 ± 0.6	0.465
sP-selectin (ng/mL)	3.2 ± 1.0	2.7 ± 1.4	0.335
Platelet aggregation (%)	59.3 ± 8.6	64.0 ± 7.5	0.209
Cotinine (ng/mL)	2.2 ± 0.7	2.2 ± 0.8	0.898

H_2_O_2_ = serum hydrogen peroxide production; NO = serum nitric oxide bioavailability; sCD40L = plasmatic levels of soluble CD40 ligand; sNox2-dp = serum soluble Nox2-derived peptide; sP-selectin = plasmatic concentration of soluble P-selectin.

**Table 3:** Chronic users of traditional combustion cigarettes.

Characteristic	Male	Female	*p*
Subjects	10	10	
Age (years)	24.7 ± 2.9	29.8 ± 5.0	0.012
Height (cm)	179.7 ± 7.0	164.3 ± 6.8	<0.001
Weight (kg)	78.6 ± 11.9	58.4 ± 6.4	<0.001
Body mass index (kg/m^2^)	24.3 ± 3.5	21.6 ± 2.0	0.046
Systolic blood pressure (mmHg)	121.5 ± 8.8	115 ± 9.7	0.135
Diastolic blood pressure (mmHg)	79.2 ± 5.8	71 ± 9.1	0.027
Mean blood pressure (mmHg)	93.3 ± 2.8	85.7 ± 8.5	0.015
Total cholesterol (mg/dL)	180.1 ± 15.4	183.1 ± 10.3	0.615
Years of smoking	4.5 ± 1.8	8.2 ± 3.5	0.009
Cigarettes per day	13.4 ± 8.0	12.8 ± 2.66	0.824
Flow-mediated dilation (%)	2.1 ± 2.9	3.5 ± 4.2	0.380
Nitric oxide (µmol/L)	11.7 ± 3.0	9.2 ± 2.5	0.060
sNox2-dp (ng/mL)	46.4 ± 4.2	41.7 ± 11.4	0.236
H_2_O_2_ (µmol/L)	36.1 ± 15.0	32.9 ± 22.9	0.720
sCD40L (ng/mL)	3.1 ± 1.0	3.5 ± 1.0	0.388
sP-selectin (ng/mL)	8.8 ± 2.8	9.9 ± 2.7	0.366
Platelet aggregation (%)	78 ± 9.9	81.2 ± 7.5	0.426
Cotinine (ng/mL)	147.6 ± 63.2	145.1 ± 24.9	0.909

H_2_O_2_ = serum hydrogen peroxide production; NO = serum nitric oxide bioavailability; sCD40L = plasmatic levels of soluble CD40 ligand; sNox2-dp = serum soluble Nox2-derived peptide; sP-selectin = plasmatic concentration of soluble P-selectin.

**Table 4:** Chronic users of heat-not-burn cigarettes.

Characteristic	Male	Female	*p*
Subjects	8	12	
Age (years)	37.3 ± 10.5	34.6 ± 10.0	0.573
Height (cm)	180.1 ± 4.0	171.7 ± 4.9	0.000
Weight (kg)	77.3 ± 8.5	59.2 ± 7.3	0.000
Body mass index (kg/m^2^)	23.8 ± 2.1	20.1 ± 2.9	0.006
Systolic blood pressure (mmHg)	117.5 ± 7.6	111.3 ± 9.8	0.145
Diastolic blood pressure (mmHg)	73.9 ± 7.0	74.8 ± 8.1	0.806
Mean blood pressure (mmHg)	88.4 ± 6.1	86.9 ± 7.9	0.656
Total cholesterol (mg/dL)	178.5 ± 18.4	166.2 ± 11.2	0.077
Smoking status			
Non-smoker	0	0	
Combustion cigarette smoker	0	0	
Heat-not-burn cigarette smoker	8	12	
Years of smoking	1.6 ± 0.4	1.3 ± 0.5	0.185
Cigarettes per day	16.3 ± 6.9	11.3 ± 4.8	0.077
Flow-mediated dilation (%)	3.9 ± 1.8	3.9 ± 3.0	0.999
Nitric oxide (µmol/L)	8.9 ± 3.3	10.9 ± 3.2	0.187
sNox2-dp (ng/mL)	37.4 ± 4.2	39.7 ± 5.1	0.305
H_2_O_2_ (µmol/L)	30.3 ± 10.2	27.7 ± 6.5	0.493
sCD40L (ng/mL)	2.8 ± 0.6	3.1 ± 0.9	0.318
sP-selectin (ng/mL)	7.1 ± 2.3	8.4 ± 3.0	0.326
Platelet aggregation (%)	76.3 ± 9.2	75.6 ± 8.3	0.868
Cotinine (ng/mL)	135.3 ± 29.6	136.2 ± 42.8	0.959

H_2_O_2_ = serum hydrogen peroxide production; NO = serum nitric oxide bioavailability; sCD40L = plasmatic levels of soluble CD40 ligand; sNox2-dp = serum soluble Nox2-derived peptide; sP-selectin = plasmatic concentration of soluble P-selectin.

**Table 5:** Multivariable regression models, with and without interaction term *.

Characteristic	Main Effects				Interactions		
	TCC vs. NS	HNBC vs. NS	HNBC vs. TCC	Female Sex	TCC vs. NS	HNBC vs. NS	HNBC vs. TCC
Flow-mediated dilation	*p* < 0.001 (−4.9 [−7.4; −2.5])	*p* = 0.002 (−4.0 [−6.5; −1.5])	*p* = 0.453 (0.9 [−1.5; 3.4])	*p* = 0.166 (1.4 [−0.6; 3.5]	*p* = 0.594	*p* = 0.273	*p* = 0.566
Nitric oxide	*p* < 0.001 (−33.3 [−37.6; −29.3])	*p* < 0.001 (−34.0 [−38.1; −29.8])	*p* = 0.816 (−0.5 [−4.7; 3.7])	*p* = 0.424 (1.4 [−2.0; 4.8]	*p* = 0.088	*p* = 0.534	*p* = 0.279
sNox2-dp	*p* < 0.001 (24.6 [20.0; 29.3])	*p* < 0.001 (19.3 [14.7; 24.0])	***p* = 0.026** **(−5.3 [−9.9; −0.7])**	*p* = 0.949 (−0.1 [−3.9; 3.7])	*p* = 0.142	*p* = 0.971	*p* = 0.136
H_2_O_2_	*p* < 0.001 (25.4 [17.7; 33.1])	*p* < 0.001 (19.9 [12.2; 27.5])	*p* = 0.154 (−5.5 [−13.2; 2.1])	*p* = 0.456 (−2.4 [−8.7; 3.9])	*p* = 0.817	*p* = 0.873	*p* = 0.945
sCD40L	*p* < 0.001 (1.8 [1.3; 2.3])	*p* < 0.001 (1.5 [1.0; 2.0])	*p* = 0.147 (−0.4 [−0.9; 0.1])	*p* = 0.130 (0.3 [−0.1; 0.7])	*p* = 0.665	*p* = 0.730	*p* = 0.932
sP-selectin	*p* < 0.001 (6.4 [4.9; 7.9])	*p* < 0.001 (4.9 [3.5; 6.4])	***p* = 0.050** **(−1.5 [−3.0; 0.0])**	*p* = 0.316 (0.6 [−0.6; 1.8])	*p* = 0.268	*p* = 0.232	*p* = 0.919
Platelet aggregation	*p* < 0.001 (17.8 [12.5; 23.1])	*p* < 0.001 (13.8 [8.5; 19.2])	*p* = 0.140 (−4.0 [−9.3; 1.3])	*p* = 0.270 (2.4 [−1.9; 6.8])	*p* = 0.786	*p* = 0.330	*p* = 0.478
Cotinine	*p* < 0.001 (144.1 [122.1; 166.2])	*p* < 0.001 (133.7 [111.6; 155.7])	*p* = 0.346 (−10.5 [−32.6; 11.6])	*p* = 0.953 (−0.5 [−18.7; 17.6])	*p* = 0.910	*p* = 0.969	*p* = 0.881

* Reported as *p* values (point estimate of effect [95% confidence interval]); HNBC = heat-not-burn cigarette; NS = non-smoker; TCC = traditional combustion cigarette; H_2_O_2_ = serum hydrogen peroxide production; NO = serum nitric oxide bioavailability; sCD40L = plasmatic levels of soluble CD40 ligand; sNox2-dp = serum soluble Nox2-derived peptide; sP-selectin = plasmatic concentration of soluble P-selectin. *p* values in bold show a statistically significant interaction (*p* < 0.05) between the HNBC and TCC groups.

## Data Availability

The data presented in this study are available on request from the corresponding author. The data are not publicly available due to privacy restrictions.
